# Honoring Professor Hugo Lorenz Obwegeser: A Visionary in Maxillofacial Surgery and Orthodontics

**DOI:** 10.7759/cureus.68420

**Published:** 2024-09-01

**Authors:** João Mendes Abreu, Bárbara R Sousa, João Oliveira, Érica Cerqueira, Pedro Cabeça Santos, Nuno S Gil

**Affiliations:** 1 Faculty of Medicine, Clinical and Academic Centre of Coimbra, Coimbra, PRT; 2 Stomatology Service - Head, Neck and Skin Surgery Department, Unidade Local de Saúde de Coimbra, Coimbra, PRT; 3 Faculty of Medicine, University of Coimbra, Coimbra, PRT; 4 Maxillofacial Service - Head, Neck and Skin Surgery Department, Unidade Local de Saúde de Coimbra, Coimbra, PRT; 5 Aesthetic Medicine, Universidad de Alcalá Madrid, Madrid, ESP; 6 Stomatology Department, Unidade Local de Saúde de São João, Porto, PRT

**Keywords:** malocclusion, orthodontics, orthognathic surgery, medical innovation, medical stories, biographies, historical vignette, historical vignettes

## Abstract

Hugo Lorenz Obwegeser was a pioneering Austrian surgeon whose contributions profoundly transformed the field of maxillofacial surgery. His groundbreaking work marked a pivotal turning point, enabling more sophisticated and effective corrections of facial deformities.

Obwegeser revolutionized his area of expertise by introducing innovative osteotomies of the mandible and maxilla, which became foundational techniques for addressing facial asymmetries. In addition to his surgical advancements, Obwegeser was a key figure in establishing the European Association for Cranio-Maxillo-Facial Surgery, helping to define the modern scope of the specialty.

His legacy in maxillofacial surgery is distinguished by his unwavering commitment to innovation, mentorship, and the continuous advancement of surgical practices. This article aims to honor the extraordinary achievements of Hugo Lorenz Obwegeser and his lasting impact on the field of maxillofacial surgery.

## Introduction and background

The concept of "there is beauty in symmetry" has long been a guiding principle for orthodontists, plastic surgeons, and maxillofacial surgeons in addressing dentofacial deformities. However, it is nearly impossible to replicate such a case in nature, including in humans [1]. Furthermore, in certain instances, an absolute degree of symmetry may be perceived as less aesthetically pleasing [2].

The etiology of facial asymmetry can be classified into three principal categories: congenital, developmental, and acquired. Moreover, it can be categorized according to the structures involved, namely skeletal, dental, and functional, with the potential for patients to be included in one, two, or all three categories [1]. Furthermore, the alignment of dental and facial skeletal structures can be analyzed based on their sagittal relationships. Thus, Class I represents an optimal standard, while Classes II and III indicate malocclusion [3]. In addition, it is well documented that patients with Class II and III malocclusions experience a reduction in their oral health-related quality of life (OHRQoL) and benefit significantly from combined orthodontic and surgical treatment when clinically indicated [4].

However, this practice was not possible until 1969 when Professor Hugo Lorenz Obwegeser became the first surgeon to undertake the simultaneous repositioning of the maxilla and mandible in a single operation. This innovation marked a pivotal turning point in the field of maxillofacial surgery, as it enabled the simultaneous surgical modification of the facial bones, thereby restoring the appropriate anatomical and functional relationships in patients with dentofacial skeletal abnormalities. Thus, it effectively transformed the existing paradigm [5,6].

## Review

Journey and education

Born in Hohenems, in the Austrian Rhine Valley, on October 21, 1920, Professor Obwegeser obtained his medical degree in general medicine in 1945, from the University of Innsbruck, Innsbruck, Austria. Subsequently, Professor Obwegeser relocated to Vienna, Austria, to join the renowned Rockitansky Institute of Pathology at the University of Vienna, enriching his academic expertise in pathology and microbiology. However, despite initially intending to pursue a career in internal medicine or obstetrics and gynecology, these ambitions ultimately did not materialize. An alternative option was presented to him in the form of a remunerated training position in maxillofacial surgery at the University Hospital in Graz, Austria, offered by Professor Richard Trauner; he commenced his exercise in the field and was associated with Professor Trauner for six years [5,6].

During this period, Professor Obwegeser also undertook a two-year formal dental training course, obtaining a formal degree in dentistry, in 1949, at the University of Graz, enabling him to gain qualifications in both specialties and to develop a comprehensive understanding of the intricate interrelationships between the oral and maxillofacial structures [5,6].

Additionally, while developing his surgical acumen, Professor Obwegeser had the opportunity to accompany Sir Harold Gillies, with whom he learned reconstructive surgery at the Plastic and Jaw unit at Rooksdowne House in London, United Kingdom, from October 1951 to February 1952. During this period, he was able to refine and apply fundamental principles of reconstructive surgery, including meticulous planning and precise handling of soft tissues. Furthermore, during his tenure at the Rooksdowne House, Professor Obwegeser had the opportunity to meet Ralph Millard and Ivo Pitanguy, two pioneering figures in the field of plastic surgery, with whom he remained in contact during his professional life [5,6].

To achieve excellence in all fields, in 1954, Professor Obwegeser perfected his training in plastic surgery by working in collaboration with Professor Eduard Schmid for six months in Stuttgart [5,6].

In the middle of the 1950s, Hugo Obwegeser was sent to Zurich by Professor Trauner to succeed the oral surgeon and establish a department of maxillofacial surgery. However, the lack of financial resources and the opposing winds from his new chief made the situation almost unbearable. Finally, in 1962, with the support of the professors of the surgical department of the university and the dental school, who wanted to keep him in Zurich, he was appointed Extraordinarius ad personam (associate professor) and chief of the department of maxillofacial surgery with six patient beds. Eight years later, in 1970, he was promoted to full professor at the medical faculty of the University of Zurich. Successively and quickly, Hugo Obwegeser expanded this department, making it one of the most prestigious maxillo-facial departments worldwide.

**Figure 1:**
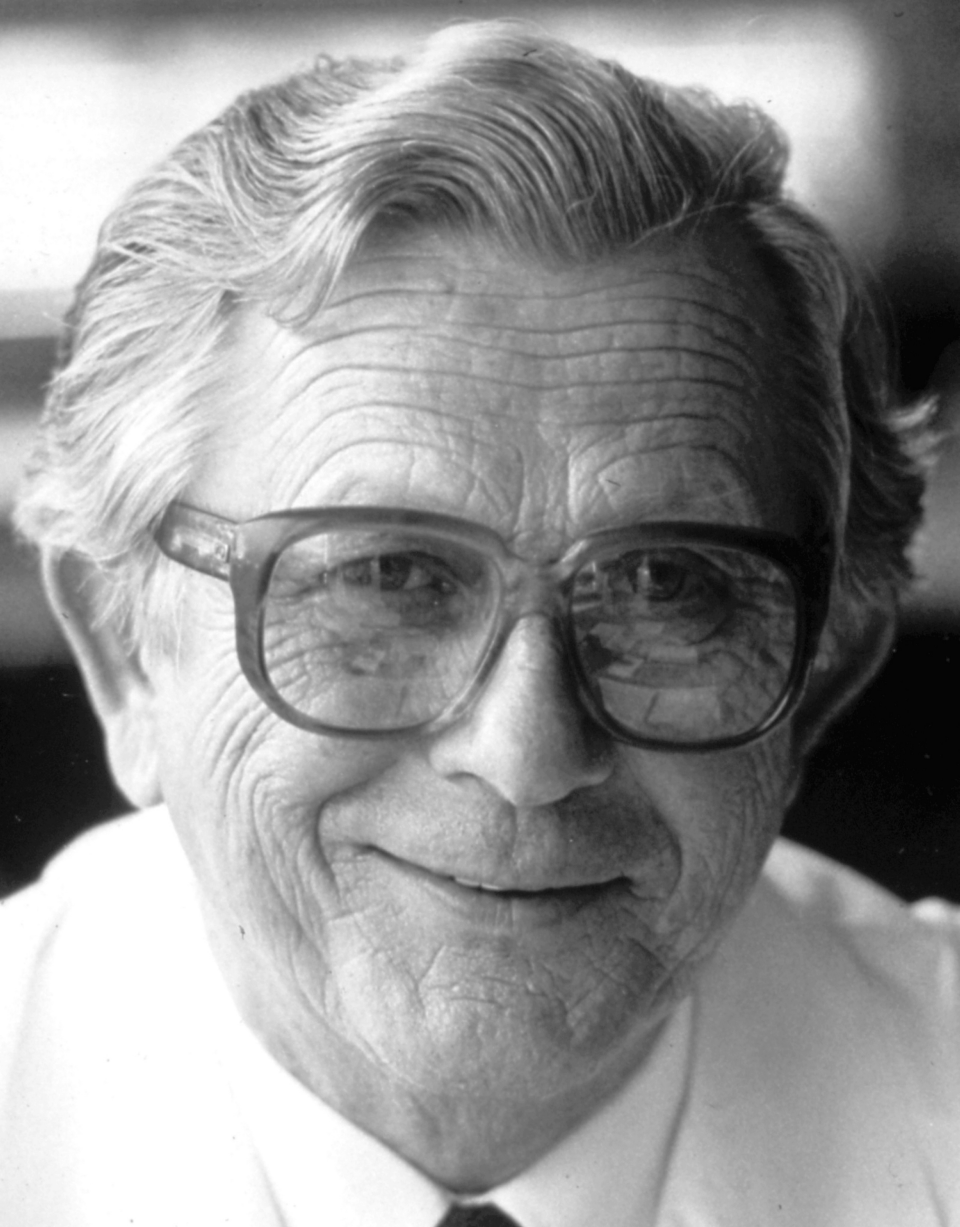
Professor Hugo Lorenz Obwegeser (1987) Part of the personal archive of Professor Joachim Obwegeser; used with permission

Professor Obwegeser retired in 1987 (Figure 1), the same year he was nominated as Professor Emeritus of the Faculty of Medicine at the University of Zurich. He then devoted the rest of his life to his family and friends in Zurich. Despite this change in circumstances, he remained an active and respected member of the medical community, attending numerous scientific meetings and lecturing regularly.

Contributions to the field of maxillofacial surgery

**Figure 2:**
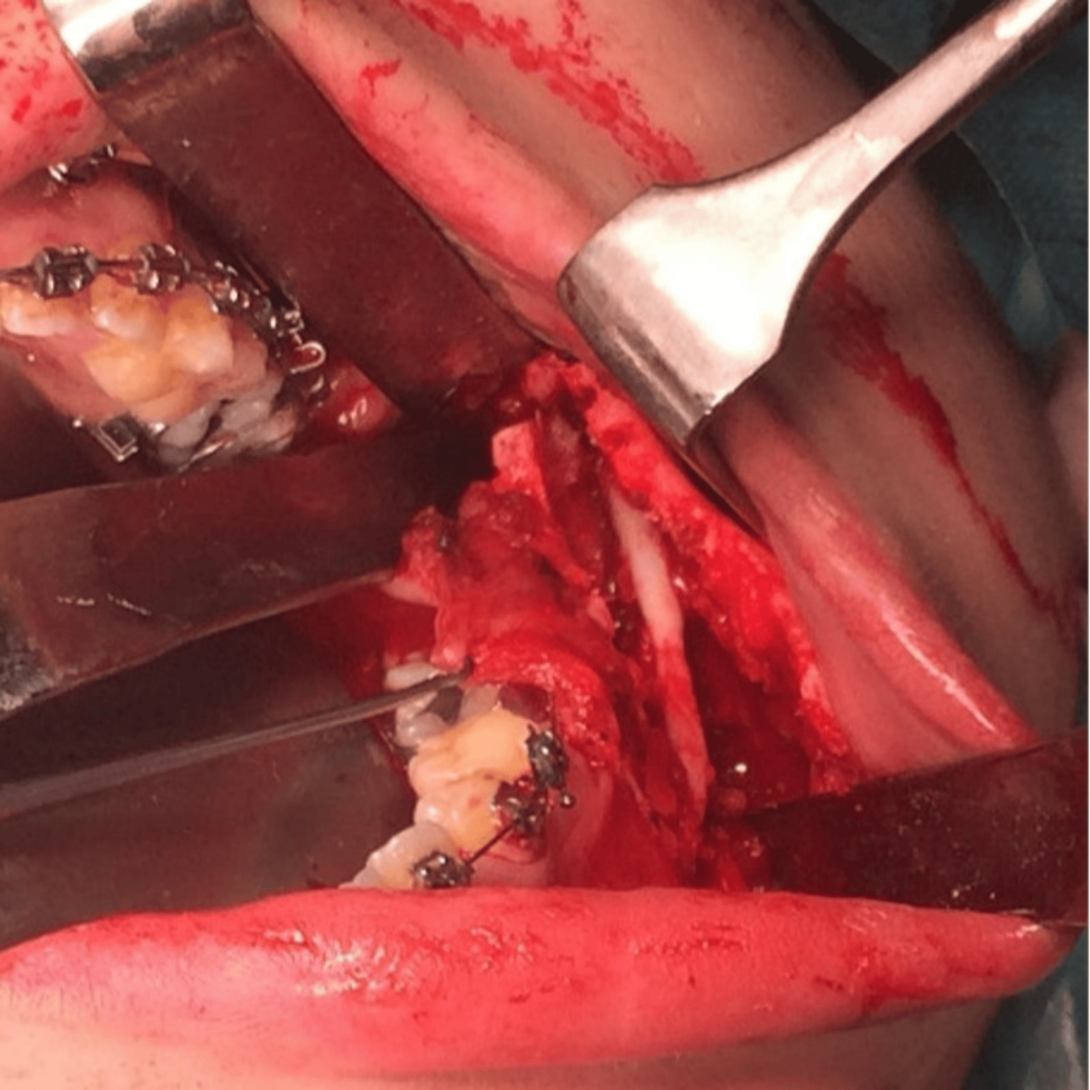
Sagittal split osteotomy of the mandible (intraoperative view) Part of the personal archive of the authors

It was not until 1953 that Professor Obwegeser first performed a novel technique he called the "sagittal splitting" osteotomy of the mandible, which he published two years later [7]. It was a new approach that allowed for the surgical correction of cases of mandibular excess, deficiency, and/or asymmetry through the performance of a posterior osteotomy of the mandible, entailing the complete separation of the bone into two/three fragments while ensuring the preservation of the inferior alveolar neurovascular bundle (Figure 2) [8].

**Figure 3:**
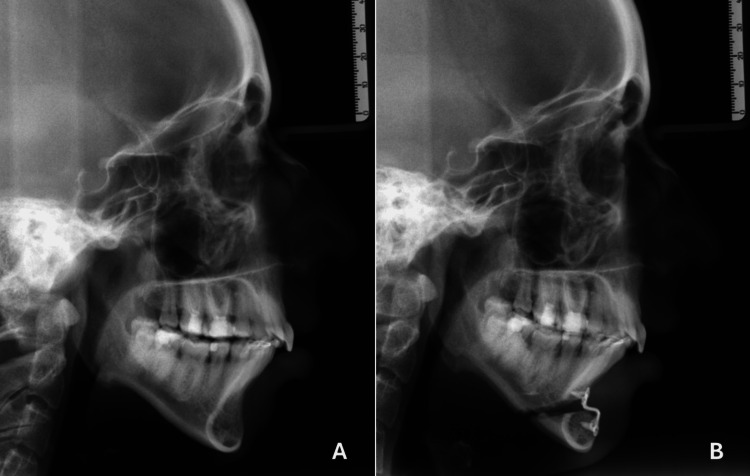
Genioplasty (radiographic view) A) Pre-operative radiographic study and B) post-operative radiographic study Part of the personal archive of the authors

This was followed, in 1957, by the performance of the first intraoral sliding osseous genioplasty in a living patient [9,10]. A technique that remains in use to this day, enabling the isolated or simultaneous correction of gnathic defects (Figure 3).

Both procedures offered the key benefit of eliminating the necessity for bone grafting in mandibular surgery. Furthermore, they provided the additional advantage of an intraoral approach, avoiding facial incisions and scarring [5,6].

**Figure 4:**
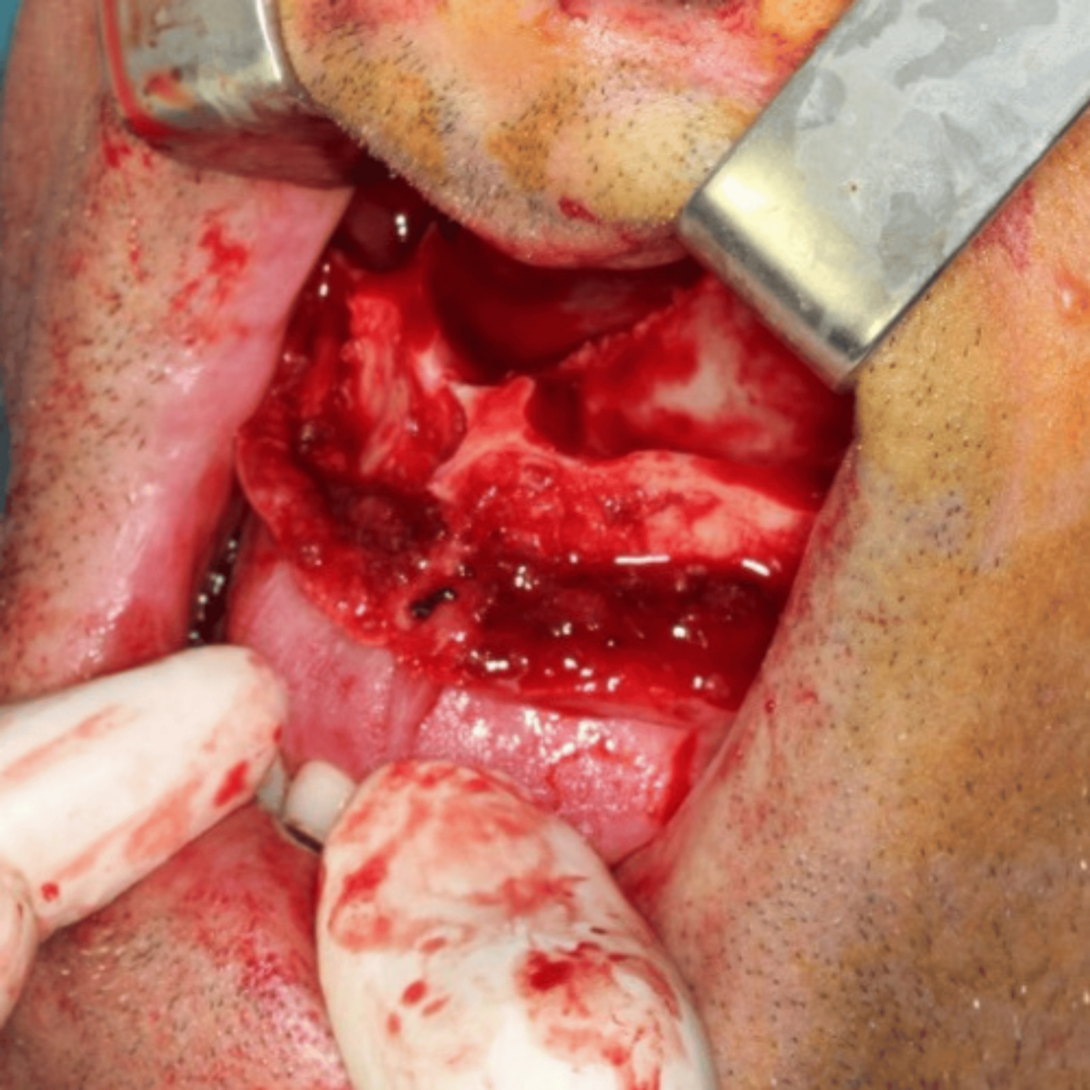
Le Fort type I osteotomy of the maxilla (intraoperative view) Part of the personal archive of the authors

Following the favorable outcomes of these techniques, Professor Obwegeser began formulating a hypothesis regarding an analogous procedure on the maxillary bone. Subsequently, in 1965, inspired by René Le Fort's work regarding complex fractures of the midface, particularly the Le Fort type I, he performed the inaugural osteotomy of the maxilla, in conjunction with pterygomaxillary disjunction, which he named following the aforementioned classification system [11]. An innovative surgical technique permitted surgeons to address midface dentofacial deformities, thereby providing them with comprehensive three-dimensional control and access to the skull base (Figure 4) [12].

**Figure 5:**
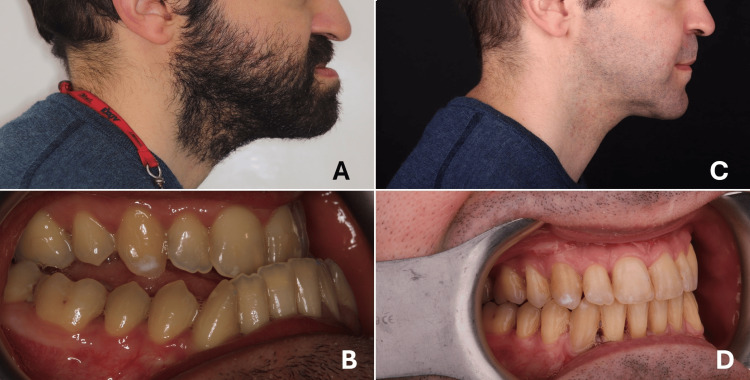
Bimaxillary orthodontic and orthognathic treatment A) Pre-treatment extraoral lateral view, B) pre-treatment intraoral lateral view, C) post-treatment extraoral lateral view, and D) post-treatment intraoral lateral view Part of the personal archive of Dr. João Pato; used with permission

These procedures established the foundation for the inaugural bimaxillary orthognathic surgery, completed two years later, in 1969 [13]. This revolutionary methodology permitted, in conjunction with orthodontists, the correction of not only malocclusion cases but also mixed and complex skeletal deformities and some of their consequences, such as obstructive sleep apnea (Figure 5) [8,12,13].

A summary of the surgical techniques developed and implemented by Professor Obwegeser is shown in Table 1.

**Table 1:** Pioneering surgical techniques developed by Professor Hugo Lorenz Obwegeser

Year	Surgical Technique	Description
1953	Sagittal split osteotomy (mandible)	A procedure that splits the mandible sagittally, allowing for intraoral surgery, avoiding facial scars.
1957	Genioplasty	A surgical procedure to alter the chin’s shape and position.
1965	Le Fort I-type osteotomy (maxilla)	A technique involving the horizontal repositioning of the maxilla to correct midface deformities.
1969	Bimaxillary osteotomy	Simultaneous repositioning of both the maxilla and mandible in one surgical procedure.

Achievements and awards

Professor Obwegeser played a pivotal role in the establishment of the European Association for Cranio-Maxillo-Facial Surgery (EACMFS), which he had the privilege to preside over. During his active life, he additionally occupied the position of editor-in-chief of the Journal of Oral and Maxillofacial Surgery, as well as president of the German Society of Oral and Maxillofacial Surgery [5,6]. He was also instrumental in the naming of the field and medical specialty, as well as establishing the importance of having a dental component in conjunction with the medical and surgical to achieve excellence in the area [5,6].

Lastly, Professor Obwegeser made a significant contribution to the scientific community, with hundreds of publications, including articles, lectures, and books, that serve as a testament to his influence and have contributed to the advancement of scientific knowledge.

The legacy of Professor Hugo Obwegeser

**Figure 6:**
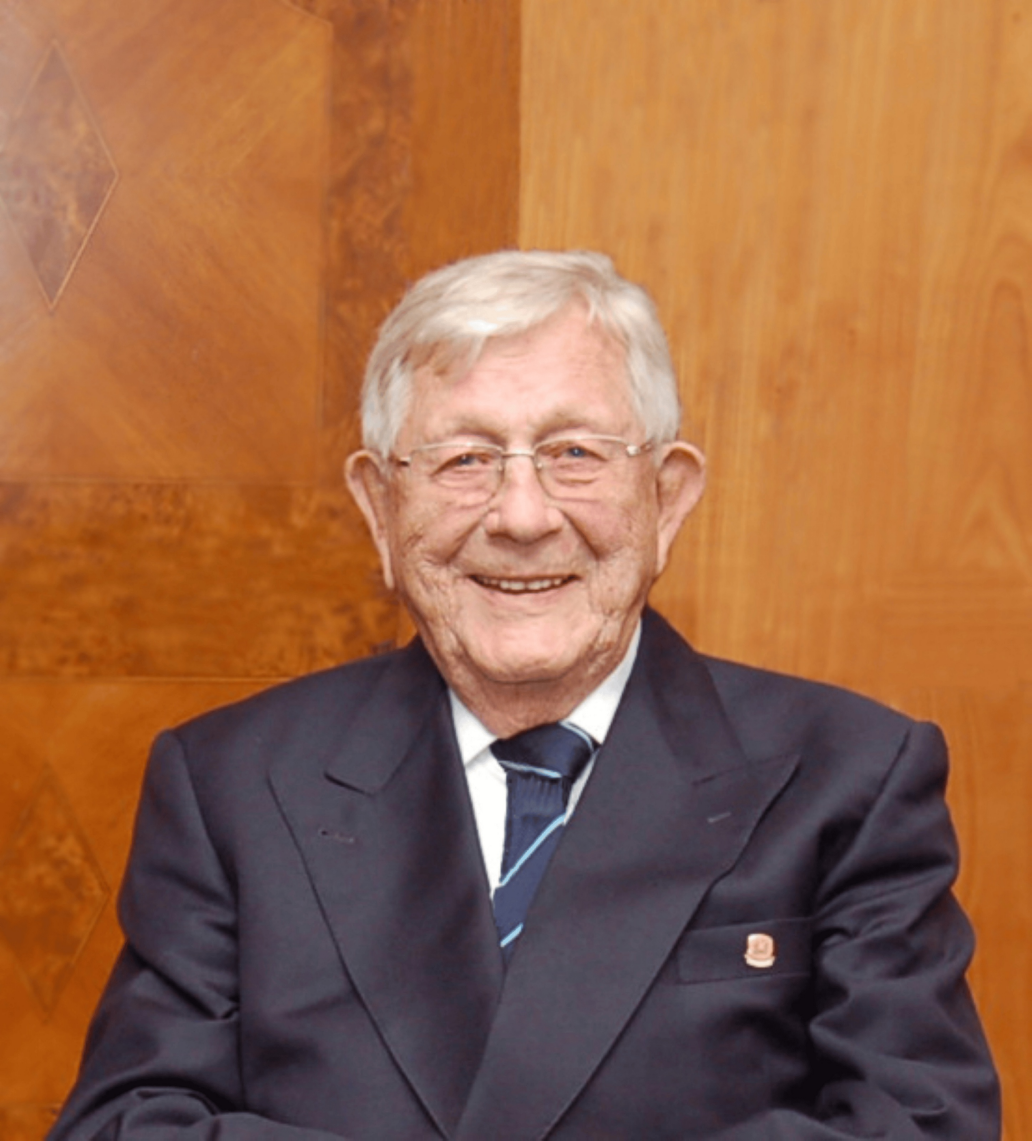
Professor Hugo Lorenz Obwegeser (2015) Part of the personal archive of Professor Joachim Obwegeser; used with permission

Professor Obwegeser (Figure 6) lived until the age of 97, passing away on September 2, 2017, in Zurich, Switzerland, in a peaceful manner [5,6].

**Figure 7:**
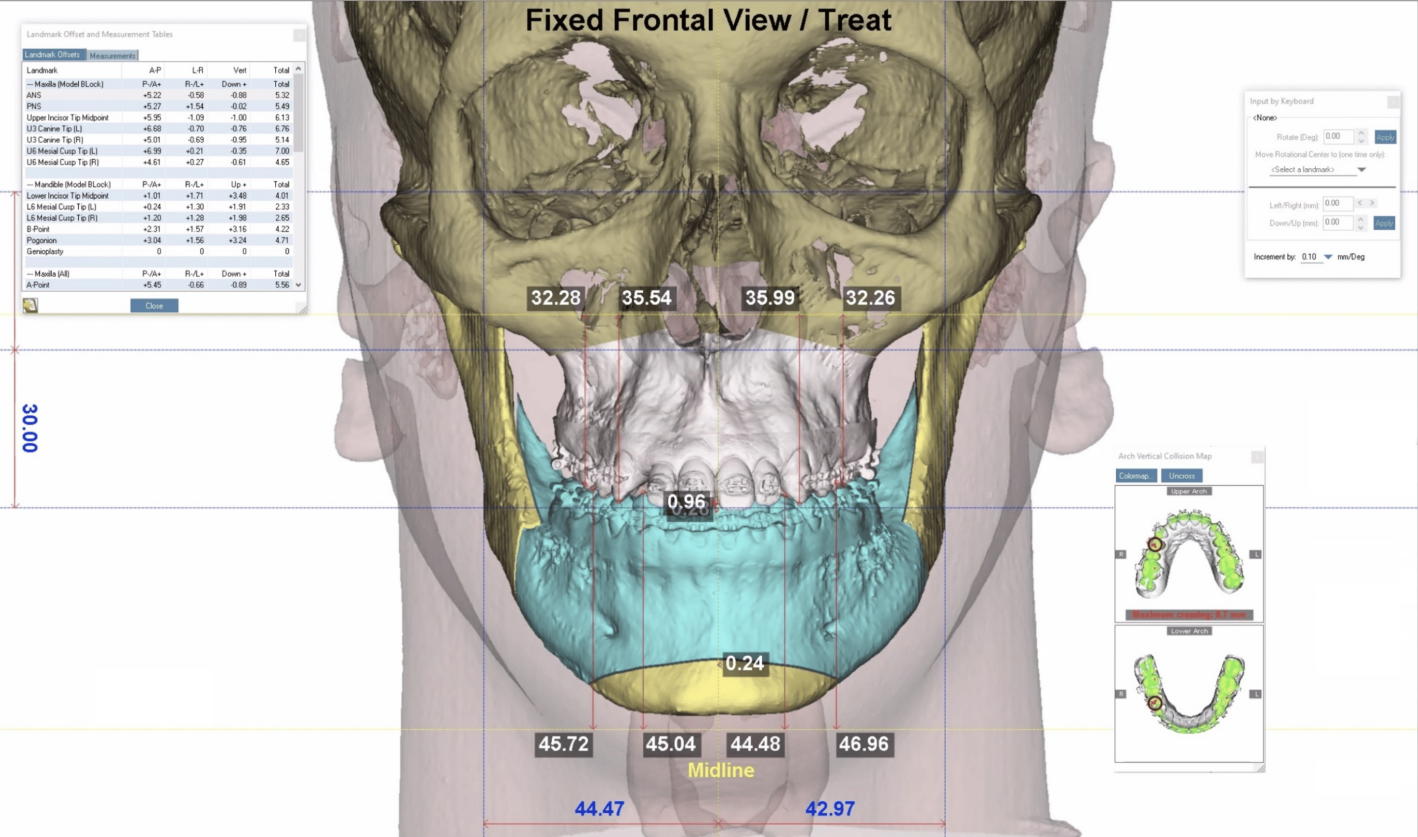
Three-dimensional planning of a bimaxillary orthognathic surgery Part of the personal archive of the authors

**Figure 8:**
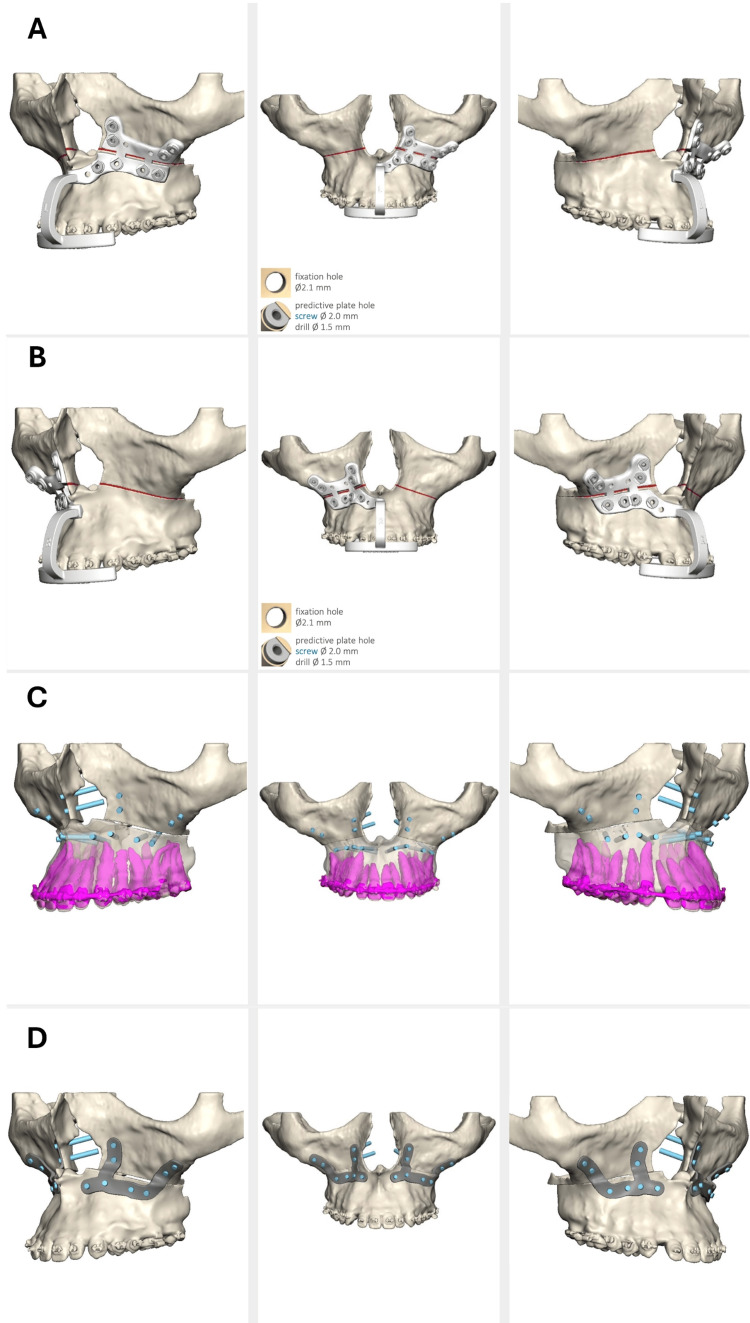
Three-dimensional planning and virtual fabrication of surgical guides and custom osteosynthesis plates A) Right maxillary surgical guide, B) left maxillary surgical guide, C) fixation screws orientation, and D) custom osteosynthesis plates Part of the personal archive of the authors

Fortunately, he was able to witness the refinement of his techniques and the technological advances that allowed for the continuous innovation of orthognathic surgery [8,12]. The foremost is the implementation of piezoelectric surgery, coupled with comprehensive digital and three-dimensional planning, allowing the use of customized surgical guides, osteosynthesis plates, and, more recently, minimally invasive techniques (Figure 7, 8) [14-19].

## Conclusions

Professor Hugo Lorenz Obwegeser was a pioneering figure in the field of medicine, unifying the disciplines of orthodontics, oromaxillofacial surgery, and/or craniomaxillofacial surgery.

During his lifetime, Professor Obwegeser not only established the foundations for the current oral and maxillofacial surgery, but also left a legacy of excellence, perseverance, and humility through his mentorship and work. Considering the enduring relevance of his teachings, we express our gratitude for his contributions, which remain as pertinent today as they were six decades ago.

## References

[1] Agrawal M, Agrawal JA, Nanjannawar L, Fulari S, Kagi V (2015). Dentofacial asymmetries: challenging diagnosis and treatment planning. J Int Oral Health.

[2] Zheng R, Ren D, Xie C, Pan J, Zhou G (2021). Normality mediates the effect of symmetry on facial attractiveness. Acta Psychol (Amst).

[3] Lombardo G, Vena F, Negri P (2020). Worldwide prevalence of malocclusion in the different stages of dentition: a systematic review and meta-analysis. Eur J Paediatr Dent.

[4] Duarte V, Zaror C, Villanueva J (2022). Oral health-related quality of life changes in patients with dentofacial deformities class II and III after orthognathic surgery: a systematic review and meta-analysis. Int J Environ Res Public Health.

[5] Kadam D (2023). Professor Hugo Lorenz Obwegeser (1920-2017): an icon of orthognathic surgery. Indian J Plast Surg.

[6] Naini FB (2017). Hugo L. Obwegeser (1920-2017) - the father of modern orthognathic surgery. J Orthod.

[7] Trauner R, Obwegeser H (1955). Zur operationstechnik bei der progenie und anderen unterkieferanomalien. Dtsch Z Mund Kieferheilk.

[8] Monson LA (2013). Bilateral sagittal split osteotomy. Semin Plast Surg.

[9] TR R, OB H (1957). The surgical correction of mandibular prognathism and retrognathia with consideration of genioplasty. I. Surgical procedures to correct mandibular prognathism and reshaping of the chin. Oral Surg Oral Med Oral Pathol.

[10] Obwegeser H (1958). Die kinnvergrößerung. Öst Z Stomat.

[11] Obwegeser H (1957). Eingriffe am oberkiefer zur korrektur des progenen zustandsbildes. Schweiz Mschr Zahnheilk.

[12] Buchanan EP, Hyman CH (2013). LeFort I osteotomy. Semin Plast Surg.

[13] Obwegeser H (1970). The one time forward movement of the maxilla and backward movement of the mandible for the correction of extreme prognathism [Article in German]. SSO Schweiz Monatsschr Zahnheilkd.

[14] Conley RS (2022). Orthognathic surgery past, present, and future. Clin Investig Orthod.

[15] Alrefai M, Daboul A, Fleischhacker B, Landes C (2022). Piezoelectric versus conventional techniques for orthognathic surgery: systematic review and meta-analysis. J Stomatol Oral Maxillofac Surg.

[16] Lee SJ, Yoo JY, Woo SY (2021). A complete digital workflow for planning, simulation, and evaluation in orthognathic surgery. J Clin Med.

[17] Francisco I, Ribeiro MP, Marques F (2022). Application of three-dimensional digital technology in orthodontics: the state of the art. Biomimetics (Basel).

[18] Espino-Segura-Illa M, Camps-Font O, Ferrer-Fuertes A, Cuesta-González F, Zubillaga-Rodríguez I, Sieira-Gil R (2024). Waferless orthognathic surgery with customized osteosynthesis and surgical guides: a prospective study. Appl Sci.

[19] AlAsseri N, Swennen G (2018). Minimally invasive orthognathic surgery: a systematic review. Int J Oral Maxillofac Surg.

